# Nav1.6 promotes inflammation and neuronal degeneration in a mouse model of multiple sclerosis

**DOI:** 10.1186/s12974-019-1622-1

**Published:** 2019-11-13

**Authors:** Barakat Alrashdi, Bassel Dawod, Andrea Schampel, Sabine Tacke, Stefanie Kuerten, Jean S. Marshall, Patrice D. Côté

**Affiliations:** 1 Department of Biology, Dalhousie University, Halifax, NS B3H 4R2 Canada; 2 Department of Biology, Al-Jouf University, Sakaka, Saudi Arabia; 3 Department of Pathology, Dalhousie University, Halifax, NS B3H 4R2 Canada; 4 Institute of Anatomy and Cell Biology Friedrich Alexander University Erlangen-Nürnberg (FAU), Erlangen, Germany; 5 Department of Microbiology and Immunology, Dalhousie University, Halifax, NS B3H 4R2 Canada; 6 Department of Ophthalmology and Visual Sciences, Dalhousie University, Halifax, NS B3H 4R2 Canada

**Keywords:** Multiple sclerosis, Inflammation, Experimental autoimmune encephalitis, Optic neuritis, Sodium channel, *Scn8a*, Nav1.6, Retinal ganglion cells, Optic nerve, Adeno-associated virus, Conditional knockout

## Abstract

**Background:**

In multiple sclerosis (MS) and in the experimental autoimmune encephalomyelitis (EAE) model of MS, the Nav1.6 voltage-gated sodium (Nav) channel isoform has been implicated as a primary contributor to axonal degeneration. Following demyelination Nav1.6, which is normally co-localized with the Na^+^/Ca^2+^ exchanger (NCX) at the nodes of Ranvier, associates with β-APP, a marker of neural injury. The persistent influx of sodium through Nav1.6 is believed to reverse the function of NCX, resulting in an increased influx of damaging Ca^2+^ ions. However, direct evidence for the role of Nav1.6 in axonal degeneration is lacking.

**Methods:**

In mice floxed for *Scn8a*, the gene that encodes the α subunit of Nav1.6, subjected to EAE we examined the effect of eliminating Nav1.6 from retinal ganglion cells (RGC) in one eye using an AAV vector harboring Cre and GFP, while using the contralateral either injected with AAV vector harboring GFP alone or non-targeted eye as control.

**Results:**

In retinas, the expression of *Rbpms*, a marker for retinal ganglion cells, was found to be inversely correlated to the expression of *Scn8a*. Furthermore, the gene expression of the pro-inflammatory cytokines *Il6* (IL-6) and *Ifng* (IFN-γ), and of the reactive gliosis marker *Gfap* (GFAP) were found to be reduced in targeted retinas. Optic nerves from targeted eyes were shown to have reduced macrophage infiltration and improved axonal health.

**Conclusion:**

Taken together, our results are consistent with Nav1.6 promoting inflammation and contributing to axonal degeneration following demyelination.

## Highlights

- Retinas from eyes subjected to selective Nav1.6 targeting have increased retinal ganglion cell survival and reduced inflammation and reactive gliosis.
- Optic nerves from eyes subjected to selective Nav1.6 targeting have reduced demyelination and axonal loss.
- Findings support the hypothesis that Nav1.6 in the EAE model of MS promotes inflammation and neuronal death.

## Introduction

Multiple sclerosis (MS) is a chronic inflammatory and neurodegenerative disorder of the central nervous system (CNS), affecting more than 2.5 million people worldwide [1, 2]. It is believed that the main trigger of the disease might be an inflammatory autoimmune response within the nervous system that causes tissue damage, including demyelination and axonal damage [3–5]. The neuroinflammation may be latent in the beginning but eventually progresses into a relapsing-remitting phase, at which point it is possible to lose and regain myelin [6]. In the early stages of the disease, axonal demyelination, with the axon remaining viable, is associated with variable degrees of inflammation and astrogliosis [7]. However, permanent neurological deficits become increasingly prominent as the neuroaxonal degeneration progresses [8].

Voltage-gated sodium (Nav) channels have been implicated in the etiology of MS and EAE as a key factor in causing axonal degeneration. The Nav1.x channel family consists of nine different pore-forming alpha-subunits (Nav1.1–Nav1.9) which assemble with two of five non-pore-forming beta-subunits (β1, β1B, β2, β3, β4). These channels are present in motor and sensory axons in the peripheral nervous system (PNS) and cluster at nodes of Ranvier in CNS axons [9]. The Nav1.6 isoform, in particular, has been associated with axonal loss following demyelination in both EAE [10, 11] and MS [12]. In axons, Nav1.6 has been shown to co-localize with the Na^+^/Ca^2+^ exchanger (NCX) and β-APP, an indicator of imminent degeneration. In addition, the co-localization between Nav1.6 and NCX was found only in axons that express β-APP, an indicator of defective transport which is commonly used as a marker of axonal damage [13]. However, in axons with damaged myelin expressing diffused Nav channels, axons expressing only the Nav1.2 isoform did not co-localize with β-APP while virtually all β-APP-expressing axons were expressing Nav1.6, along with or without Nav1.2 [11].

In this study, we examined how deleting Nav1.6 from a population of retinal ganglion cells in experimental autoimmune encephalomyelitis (EAE) mice, a common animal model of MS [3, 14] affects disease progression. Intra-animal comparisons revealed enhanced RGC survival, reduced inflammation, and improved axonal health in the Nav1.6-targeted eye versus the control eye. Taken together, our data support the hypothesis that Nav1.6 contributes to the pathophysiology of EAE, and by extension of MS and possibly other neurodegenerative disorders.

## Materials and methods

### Mice

A total of 36 mice were used in this study: C57BL/6 (Charles River, Saint-Constant, QC) (*n* = 16) and *Scn8a*^flox/flox^ homozygous for alleles of *Scn8a* harboring loxP sequences flanking the first exon (*n* = 20; a generous gift of Dr. Miriam Meisler, University of Michigan, USA [15]). Mice were housed in groups of 3 to 5 under a 12-h light/dark cycle with free access to food and water in HEPA ventilated cages at the Carleton Animal Care Facility (CACF) at Dalhousie University. All animal procedures were completed in accordance with animal care guidelines established by the Canadian Council on Animal Care and in accordance with the ARVO Statement for the Use of Animals in Ophthalmic and Vision Research. Protocols were reviewed and approved by the Dalhousie University Committee on Laboratory Animals (protocol nos. 17-012 and 19-050).

### Intravitreal injection of AAV

**Fig. 1:**
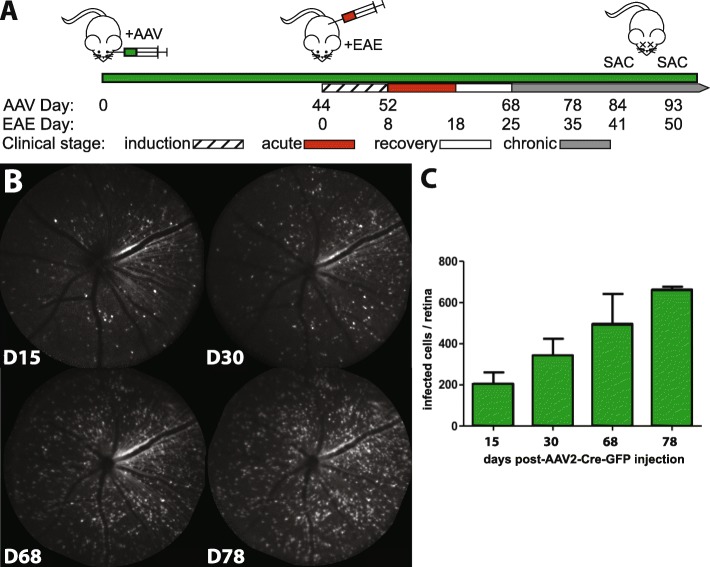
Experimental timeline and AAV transduction of inner retinal cells. **a** Experimental timeline with intravitreal injection of the AAV2-Cre-GFP or AAV2-GFP virus (+AAV), as well as the induction and clinical stages of EAE in *Scn8a*-flox mice. **b** Representative confocal scanning laser ophthalmoscopy (CSLO) images of GFP-labeled inner retinal cells in a single animal up to 78 days post-AAVCre injection. **c** Quantification of AAV transduction progression (*n* = 4)

Adeno-associated virus serotype 2 (AAV2) has been shown to preferentially target ganglion cells in the retina [5]. Using a 31 gauge needle and 10 μl syringe (Hamilton Company, Reno, NV, USA), we have intravitreally injected the left eye of 7-week-old *Scn8a*^flox/flox^ (*n* = 11; Fig. 1a day 0; 1.5 μl of 5 × 10^12^ viral genome copy number per milliliter) harboring the Cre-recombinase and enhanced GFP (eGFP, cat. no. SL100814, Signagen Laboratories, Rockville, MD) under the control of the cytomegalovirus (CMV) promoter in the left eye and the fellow eye was injected with AAV2-GFP alone (*n* = 4; cat. no. SL100812, Signagen Laboratories) or left non-injected (*n* = 7). The injection site was located posterior to the super temporal limbus and the injection was performed at a depth of approximately 1 mm. This procedure was performed in a biocontainment room under ketamine/xylazine anesthesia (ketamine, 100 mg/kg body weight; xylazine, 10 mg/kg body weight). GFP-production was used as a marker of AAV2 transduction of RGCs and was visualized in vivo by confocal scanning laser ophthalmoscopy (CSLO) before and after EAE induction (measured at days 15, 30, 68, and 78 after AAV2 injection; Fig. 1a).

### EAE induction and clinical score assessments

EAE was induced in 18–24 g female mice aged 10 to 12 weeks (total *n* = 22). C57BL/6 (*n* = 10) and in *Scn8a*^flox/flox^ mice (*n* = 12) were immunized for EAE induction, while C57BL/6 (*n* = 6) and *Scn8a*^flox/flox^ (*n* = 3) were left untreated as controls. EAE mice were injected subcutaneously with 200 μl of myelin oligodendrocyte glycoprotein (MOG_35–55_) peptide solution suspended in complete Freund’s adjuvant (CFA) with a concentration of 2 mg/ml (kit EK-2110, Hooke Laboratories, Lawrence, MA) [16]. Pertussis toxin (200 ng per mouse dissolved in PBS) was injected intraperitoneally on the day of immunization and after 2 days. Mice were monitored daily for weight changes and for clinical signs of EAE, and all scoring was done after removing cage cards by persons unaware of the animal groups as described by Miller et al. [17]. Scoring was performed according to the following criteria: (1) flaccid tail; (2) hindlimb weakness and poor righting ability; (3) inability to right and paralysis in one hindlimb; (4) both hindlimbs paralyzed with or without forelimb paralysis and incontinence; (5) moribund. Mice that reached a score of 4 before the end of the study (41 or 50 days post-EAE) were sacrificed and discarded from the study. The mice included in the study displayed a clinical score between 2.5 and 3.5, and were sacrificed in the chronic phase at 41 days (*n* = 8) or 50 days (*n* = 4) post EAE induction.

### In vivo imaging

GFP-producing RGCs were visualized by confocal scanning laser ophthalmoscopy (CSLO; Spectralis HRA, Heidelberg Engineering, Germany) at days 15, 30, 68, and 78 post AAV2 injection according to Smith and Chauhan [18]. Briefly, mice were anesthetized with an initial induction of 3–4% isoflurane (vol) and the eyes were dilated with topical mydriatics (1% tropicamide and 2.5% phenylephrine hydrochloride, Alcon Canada Inc., Mississauga, ON). Corneal hydration was maintained with ophthalmic liquid gel (Novartis Pharmaceuticals Canada Inc., Dorval, QC, Canada) and a contact lens (Cantor and Nissel, Brackley, UK). CSLO imaging was performed for each animal with an auxiliary + 25 diopter lens attached to the camera objective. Baseline images focused at the level of the nerve fiber layer were first acquired with infrared (820 nm) illumination. The camera adjusted to obtain the optimal fluorescence images (488 nm excitation, 500–550 nm emission bandpass filter) at the GCL layer. Each image was taken averaged 16 times using automatic real-time eye-tracking software.

### Immunohistochemistry

To quantify and visualize RGCs, the whole-mount retinas were incubated for 6 days at 4 °C with primary antibody against the mouse RNA-binding protein with multiple splicing (RBPMS; 1:1000 dilution, guinea pig anti-RBPMS, PhosphoSolutions, Aurora, CO, USA), which is uniquely expressed in RGCs [19]. This was followed by incubation with 1:400 Alexa Fluor® 488 conjugated rabbit anti-GFP (Molecular Probes, Eugene, OR, USA) and Cy3 conjugated donkey anti-guinea pig secondary antibody (Jackson Immuno Research Laboratories Inc., West Grove, PA, USA) overnight at 4 °C. After that, the retina was rinsed in PBS for 10 min, then incubated in the nuclear counterstain TO-PRO-3 iodide (Thermo Fisher Scientific, Waltham, MA) for 15 min. Retinas were flattened with RGCs facing up, mounted with anti-fade fluorescent mounting medium (Sigma-Aldrich, St. Louis, MO), and coverslipped. Images were taken using a × 20 objective with a confocal microscope (Nikon C1, Nikon Canada Inc., Toronto, ON). Three images with an area of 330.32 × 330.32 μm from each retina were used for RGC quantification: near the optic disk, near the periphery and at an intermediate distance. Image J was used to perform RGC counts.

### Hematoxylin and eosin staining of optic nerve

Optic nerves were collected from mice, embedded in paraffin, and sectioned longitudinally. The sections were dehydrated for 2 h at room temperature, after that fixed for 10 min with 4% paraformaldehyde (PFA), dehydrated for 2 min by a series of graded ethanol solutions, incubated for 5–7 min in hematoxylin, and transferred to distilled water. The sections were incubated for 1 min in eosin, dehydrated in gradient ethanol series, and mounted. Images were captured using a transmitted light microscope and analyzed with AxioVision 4.7 software (Carl Zeiss, Jena, Germany). The average number of cell nuclei per mm^2^ was determined for each optic nerve.

### Electron microscopy

Mice were sacrificed during the chronic phase of EAE at day 41 (*n* = 7) and day 50 (*n* = 4), and optic nerve tissue was harvested from both groups of mice C57BL/6 and “floxed” alleles of *Scn8a*. Tissues were processed as described by Kuerten et al. [20]. Tissues were fixed overnight in 2.5% glutaraldehyde in 0.1 M sodium cacodylate buffer, rinsed with 0.1 M sodium cacodylate buffer, fixed for 2 h with 1% osmium tetroxide, and then rinsed quickly with distilled water. Samples were then placed in 0.25% uranyl acetate at 4 °C, dehydrated in graded acetone solutions, embedded with Epon Araldite resin and placed in a 60 °C oven for 48 h to harden. The samples were sectioned transversally using an ultra-microtome (Reichert Ultracut R, Leica, Germany) at a thickness of 50 nm. Images were captured on a Zeiss 906 electron microscope (Carl Zeiss NTS GmbH, Oberkochen, Germany) equipped with a digital EM camera. To demonstrate the extent of the axonal loss and myelin pathology we measured the g-ratio by dividing the axon diameter by the diameter of the myelinated nerve fiber [16, 21]. Only axonal g-ratios three standard deviations above (remyelinating) or below (demyelinating) the average of the non-EAE reference group were counted. Axonal damage, including axolytic axons and neurofilament pathology, was determined qualitatively. A person unaware of the nature of the samples performed the analysis.

### Quantitative reverse-transcription polymerase chain reaction

Following euthanasia, samples were quickly removed from the mice and submerged in RNA later (Qiagen, Hilden, Germany). Total RNA was extracted using RNeasy Plus Mini Kit (Qiagen) according to the manufacturer’s instructions. Concentration of RNA samples were measured using an Epoch spectrophotometer and Take3™ Micro-Volume Plate (Biotek, VT, USA). The ratio of 260/280 was used to evaluate the purity of RNA samples. RNA samples were reverse transcribed to cDNA using QuantiTect® Reverse Transcription Kit (Qiagen). The resulting cDNA samples were diluted 1:4 and used in qRT-PCR with the primer sets from Table 1 to measure the expression of mRNAs.

**Table 1:** qPCR primers

Gene	Primer sequence or company (catalog number)
*Hprt*	Bio-Rad (cat. no. 10025636)
*Gapdh*	Bio-Rad (cat. no. 10025637)
*Il6*	Qiagen (cat. no. PPM03015A)
*Gfap*	Forward (5′-3′) AGGGGCAGATTTAGTCCAAC Reverse (5′-3′) AGGGAGTGGAGGAGTCATTC
*Scn8α*	Forward (5′-3′) GCAAGCTCAAGAAACCACCC Reverse (5′-3′) CCGTAGATGAAAGGCAAACTCT
*Rbpms*	Forward (5′-3′) AGAGCTGTACCTGCTCTTCAGACC Reverse (5′-3′) GCCTCTGCTTCTGAGCGACTGTC
*Ifng*	Bio-Rad (cat. no.10025636)

### Flow cytometry

Optic nerves were harvested, and the cells were dissociated by passing the tissues through metallic mesh followed by enzymatic digestion using 10 μg/ml collagenase D and 100 μg/ml DNase I. Single-cell suspensions were incubated with antibodies to define various types of immune cells, such as macrophages (F4/80+/CD11b+, 1:300 dilution Bioscience, USA). Cells were then washed using flow cytometry wash buffer (PBS supplemented with 1% BSA). Stained samples were harvested using BD FACS Canto™ II (BD Life Sciences, San Jose, CA, USA). All analysis and gating were done using BD FACS Diva software and FlowJo V10.2.

### Statistics

Statistical analyses were performed using a paired Student’s *t* test. Error bars represent the standard error of the mean (SEM). GraphPad Prism software was used for statistical analyses (Ver. 5.0, GraphPad Software, La Jolla, CA, USA). *P < 0.05, **P < 0.01.

## Results

In MS and EAE, the clinical symptoms are associated with and caused by the progression of the axonal degeneration [8, 22]. To test the role of Nav1.6 in axonal degeneration, we used mice that have the first exon of the Nav1.6 gene, *Scn8a*, flanked between two LoxP sites, i.e., “floxed” [15], which allows the gene to be knocked out locally in the presence of Cre-recombinase. A recombinant adeno-associated virus serotype 2 (AAV2), which preferentially targets ganglion cells in the retina, was used to deliver an expression vector containing Cre-GFP (AAVCre) or, as a control, GFP alone (AAVGFP) under the control of a cytomegalovirus promoter (Fig. 1a). Subsequent to intravitreal injection of the virus, GFP labeling was detectable in vivo by CSLO fluorescence imaging and the transduced cells were counted at 15, 30, 68, and 78 days post AAVCre injection (Fig. 1b, c). Smith and Chauhan (2018) reported that AAV2 transduction of inner retinal cells stabilizes at 35 days; consequently, it was estimated that near-maximal RGC transduction would be attained by 44 days post-AAV2 injection and this time was chosen to induce EAE. Interestingly, the number of transduced cells continued to increase at days 68 (EAE day 25) and 78 (EAE day 35). The clinical symptoms of EAE-induced mice started to appear 8 days post-immunization, and all mice (C57BL/6 and flox mice) subjected to EAE displayed a typical clinical course with the loss of body weight and motor impairment (Additional file 1: Figure S1).

**Fig. 2:**
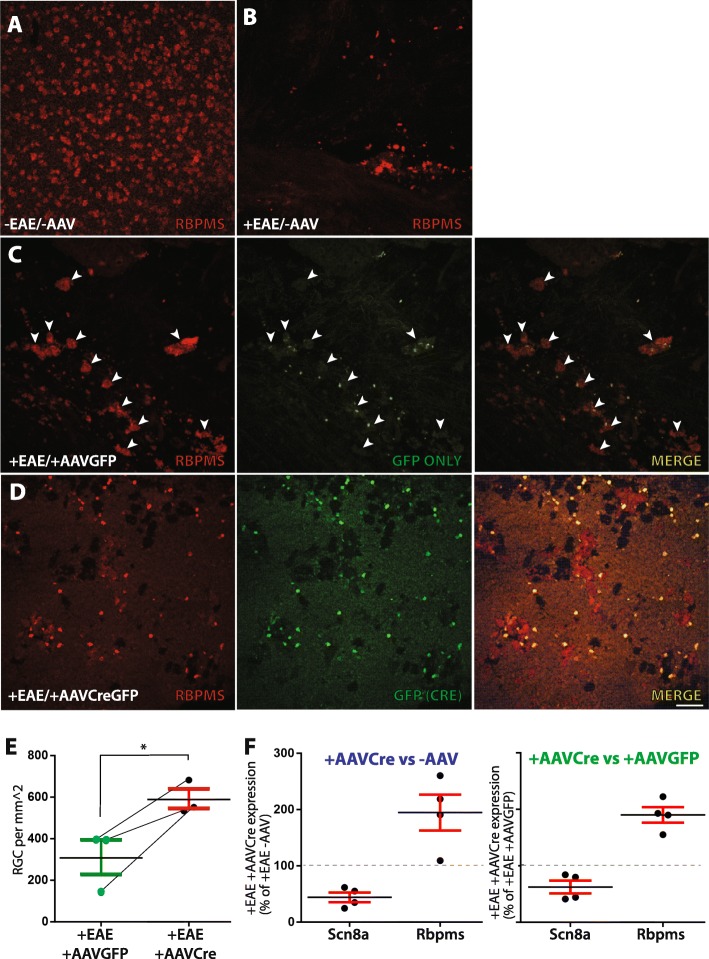
Chronic stage EAE mice have increased RGC survival in retinas with reduced *Scn8a* (Nav1.6). **a** A population of RGCs (RBPMS-positive) in a normal (−EAE/−AAVCre) retina is shown in comparison to **b** a representative image of an uninjected (−AAV) EAE mouse, and **c** a representative image of a EAE mouse retina from a control AAVGFP-treated eye (+EAE/+AAVGFP) showing RBPMS-positive degenerating RGCs (white arrowheads) with GFP occasionally co-localizing with cell remnants. **d** A representative image of an EAE mouse retina from an AAVCre-treated eye (+EAE/+AAVCreGFP) showing normal appearing GFP-positive RGCs. **e** RGC quantification in +EAE retinas treated with AAVGFP (*n* = 3) or AAVCreGFP (*n* = 3). Lines link data points for retinas from the same animal. **f** Percent of expression change for *Scn8a* and *Rbpms* in AAVCre-treated (+EAE/+AAVCreGFP; *n* = 4) eyes relative to their contralateral control uninjected (+EAE/−AAV; *n* = 4) or GFP (+EAE/+AAVGFP; *n* = 4) eye. Scale bar = 50 μm. Data are presented as the mean ± SEM. *P ≤ 0.05, paired *t* test

The effect of targeting *Scn8a* the RGC population in chronic-phase EAE, was assessed by immunostaining flat-mount retinas against RBPMS, a highly specific marker of RGCs [23]. Control non-EAE/non-AAV-treated mice (−EAE/−AAV, Fig. 2a) exhibited a dense population of RGCs, while +EAE/non-AAV-treated retinas (+EAE/−AAV, Fig. 2b) exhibited massive cells loss. EAE retinas from eyes intravitreally injected with a control GFP vector (+EAE/+AAVGFP, Fig. 2c) also revealed extensive RGC loss. In these control GFP retinas, speckled GFP staining was observed, which occasionally co-localized with enlarged and degenerating RGC soma. However, the examination of +EAE/+AAVCre retinas (*n* = 5; 3 images per retina) did not reveal any normal-appearing RGC soma that was devoid of strong “mirror-image” GFP staining. The extent of the cell loss in +EAE/+AAVGFP control is quantified in (Fig. 2e) and corresponds to 307.7 ± 83.5 cells/mm^2^ (*n* = 3) vs 3633 ± 431.3 (*n* = 3) cells/mm^2^ in non-EAE/−AAV controls. In +EAE/+AAVCre mice (Fig. 2d) large RGC loss was also observed but to a lesser extent than in contralateral +AAVGFP control retinas (589.2 ± 47.0; *p* = 0.0346; *n* = 3; Fig. 2e).

To determine the extent to which AAVCre impacted the expression of Nav1.6 and RGC survival, we compared the expression of *Scn8a* (the gene that encodes the α subunit of Nav1.6) and Rbpms (RBPMS) in retinas of EAE mice from AAVCre-injected eyes against, within the same animal, either the AAVGFP-treated or the non-injected contralateral eyes (Fig. 2f). *Scn8a* expression in AAVCre-injected retinas was reduced to 44.8% ± 8.62 of levels found in non-injected contralateral retinas (*n* = 4) and to 62.43% ± 11.38 of levels found in AAVGFP-injected contralateral retinas (*n* = 4). In the same samples, Rpbms expression was on the other hand increased to 194.8% ± 31.91 of levels found in non-injected contralateral retinas and to 190.1% ± 13.81 of levels found in AAVGFP-injected contralateral retinas. Since the AAVCre-injected eyes displayed a similar effect relative to non-injected or to +AAVGFP contralateral control eyes, we combined the two groups for subsequent analysis (referred to −AAVCre).

**Fig. 3:**
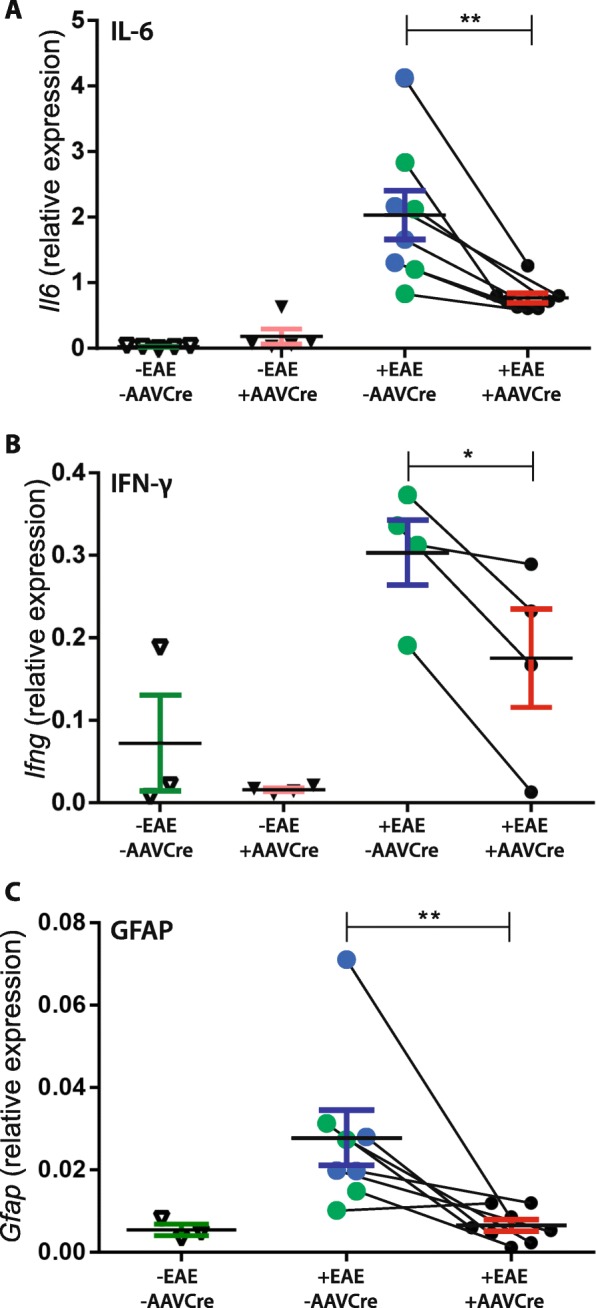
Nav1.6 promotes inflammation in EAE mice**.** Expression in the retina of the markers of inflammation. **a** *Il6* (gene that encodes IL-6) and **b** *Ifng* (IFN-γ) is compared between untreated (−EAE) or EAE-induced (+EAE) mice. The eyes of untreated (−EAE) mice are either left uninjected (−AAVCre, open triangles) or injected with AAVCreGFP (+AAVCre, closed triangles). In the EAE-induced mice, a comparison is made between AAVCreGFP-injected (+AAVCre, black dots) and the contralateral eye, which is either left uninjected (blue dots) or injected with a GFP-only control (AAVGFP, green dots). **c** Analysis of the marker of reactive gliosis *Gfap* (Glial Fibrillary Acidic Protein). Lines link data points for retinas from the same animal. Data are presented as the mean ± SEM. *P ≤ 0.05, **P ≤ 0.01, paired *t* test

To assess the role of Nav1.6 in stimulating inflammation in EAE retina, we performed real-time PCR analysis for *Il6* (IL-6), *Ifng* (IFN-gamma), *Tnf* (TNF) pro-inflammatory cytokines, the *Il10* anti-inflammatory cytokine, and *Gfap* (GFAP), a marker for reactive gliosis. The expression of *Tnf* and *Il10* was below the threshold of detection in all conditions (not shown) and the expression of *Il6*, *Ifng*, and *Gfap* in non-EAE mice was negligible to low (Fig. 3a–c). *Il6* was found to be significantly reduced (*p* = 0.0022) in all +EAE/+AAVCre (0.7697 ± 0.07507, *n* = 8) relative to contralateral control retinas (2.031 ± 0.3726, −AAVCre, *n* = 8; Fig. 3a). In addition, the expression of *Ifng* was significantly reduced (*p* = 0.0186) in +EAE/+AAVCre (0.1753 ± 0.05959; *n* = 4) versus +EAE/+AAVGFP control retinas (0.3032 ± 0.03948; *n* = 4; Fig. 3b). *Gfap* was also significantly reduced (*p* = 0.0080) in +EAE/+AAVCre (0.006452 ± 0.001426; *n* = 8) in comparison to contralateral control retinas (0.02773 ± 0.006676; −AAVCre, *n* = 8; Fig. 3c).

**Fig. 4:**
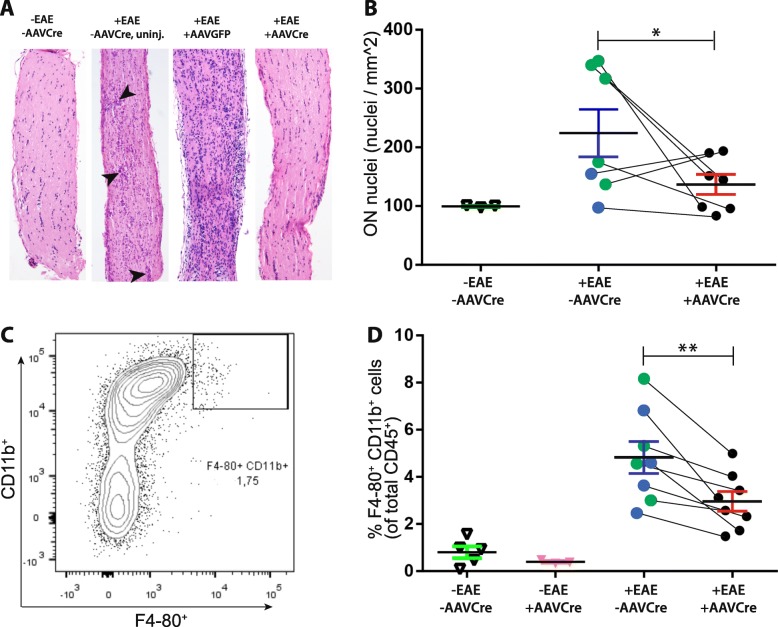
Targeting of Nav1.6 results in reduced infiltration of myeloid cells in EAE optic nerves. **a** Hematoxylin and eosin-stained optic nerves from control non-EAE-induced/uninjected eye (−EAE/−AAVCre) and from EAE-induced (+EAE) mice with eyes left uninjected (−AAVCre, uninj), injected with AAVGFP (+AAVGFP), or injected with AAVCreGFP (+AAVCre). Arrowheads indicate cellular aggregates. **b** Quantification of optic nerve nuclei from non-EAE-induced/uninjected eyes (−EAE/−AAVcre, open triangles) and from EAE-induced mice where a comparison is made between AAVCreGFP-injected (+AAVCre, black circles; *n* = 7) and the contralateral eye, which is either left uninjected (blue circles; *n* = 2) or injected with AAVGFP (green circles; *n* = 5). **c** Representative dot plot flow cytometry analysis showing the gating strategy used to identify the macrophage population expressing marker CD11b^+^ F4–80^+^ from a population of CD45^+^ cells isolated from optic nerves. **d** Flow cytometry analysis for CD11b^+^ F4–80^+^ macrophages. Lines link data points for retinas from the same animal. Scale bar, 500 μm. Data are presented as the mean ± SEM. *P ≤ 0.05, **P ≤ 0.01, paired *t* test

We then performed a histological examination of the optic nerves and found increased cell infiltration in +EAE non-injected or AAVGFP controls relative to naïve −EAE/−AAVCre with cell clusters commonly visible (indicated by arrowheads in Fig. 4a). AAVCre-treated retinas, on the other hand, had reduced cell infiltration (Fig. 4a, b). The total number of optic nerve nuclei was significantly lower (*p* = 0.0492) in +EAE/+AAVCre (132.4 ± 16.54; *n* = 7) versus control +EAE/−AAVCre mice (220.0 ± 41.91; *n* = 7; Fig. 4a, d).

The number of infiltrating macrophages, determined by flow cytometry as the percentage of F4–80^+^, CD11b^+^ of total CD45^+^ cells, was found to be similar in −EAE/+AAVCre and in −EAE/−AAVCre (Fig. 4d). The level of optic nerve infiltrating macrophages was found significantly reduced (*p* = 0.0015) in +EAE/+AAVCre (2.958 ± 0.4188; *n* = 8) vs + EAE/−AAVCre (4.818 ± 0.6789; *n* = 8; Fig. 4d).

**Fig. 5:**
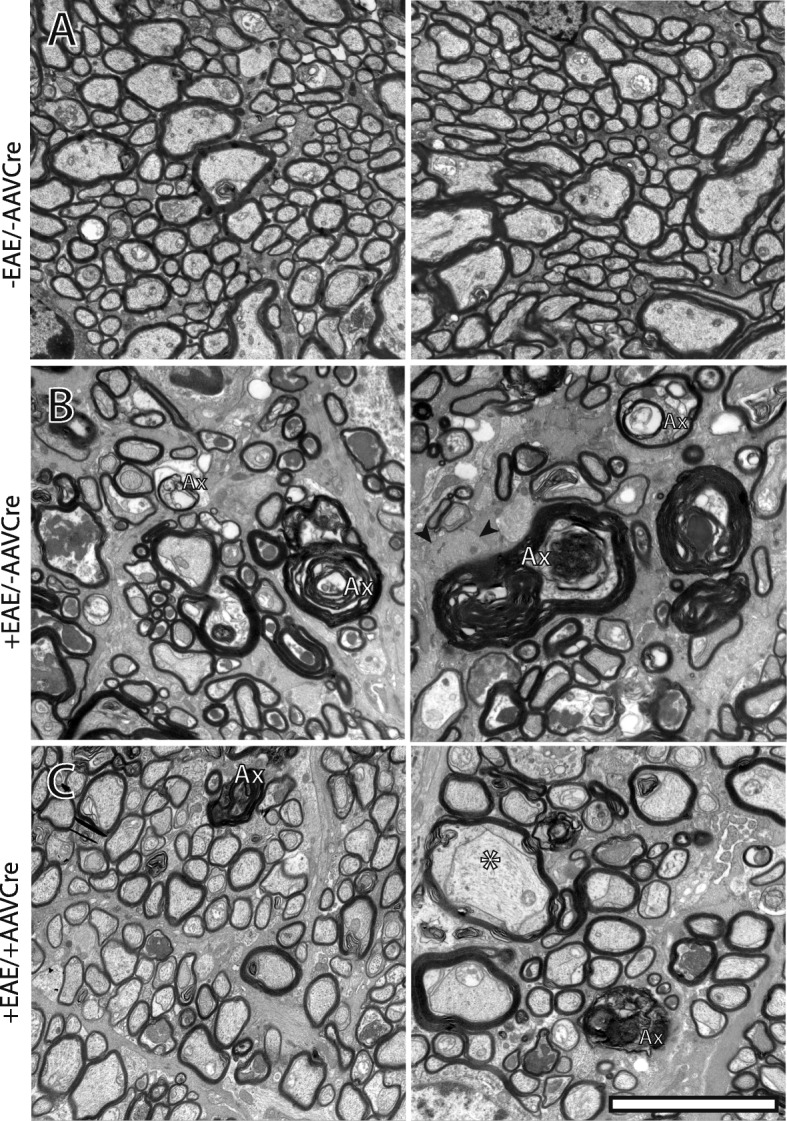
The axonal pathology is improved in optic nerves with reduced Nav1.6 levels. Representative ultra-thin transversal sections of optic nerves were obtained from control non-EAE-treated/uninjected mice (**a**, −EAE/−AAVCre), EAE-treated/uninjected (**b**, +EAE/−AAVCre), and EAE-treated/AAV2Cre-injected (**c**, +EAE/+AAVCre). Images in **b** and **c** are of retinas from the same animal. Ax, axolytic; *, demyelinating. Scale bar, 5 μm

Next, myelin and axonal pathology were assessed by comparing electron micrographs of optic nerve transversal sections from naïve (−EAE/−AAVCre, Fig. 5a), to +EAE/−AAVCre (Fig. 5b) and + EAE/+AAVCre (Fig. 5c) optic nerves. We found that axon density was severely reduced in +EAE/−AAVCre optic nerves compared to naïve optic nerves and pathological features such as demyelinating, demyelinated, and axolytic axons were frequently observed. In comparison, the axon density in +EAE/+AAVCre optic nerves was visibly increased and pathological features were less common.

**Fig. 6:**
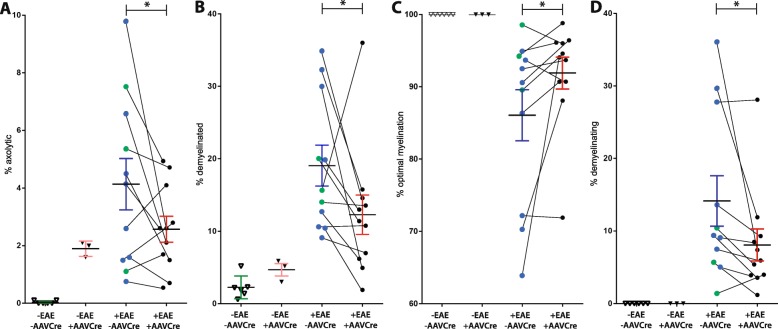
Reduced Nav1.6 levels in optic nerve is associated with decreased demyelination and reduced axonal damage. Electron micrographs of optic nerves from control non-EAE-induced (−EAE) or EAE-induced (+EAE) mice were analyzed. The non-EAE-induced optic nerves are from eyes either left uninjected (−AAVCre, open triangles) or injected with AAVCreGFP (+AAVCre, closed triangles). In the EAE-induced mice, a comparison is made between AAVCreGFP-injected (+AAVCre, black circles) and the contralateral eye, which is either left uninjected (blue dots) or injected with a GFP-only control AAV (green circles). We examined the frequency of **a** axolytic axons and **b** demyelinated axons (axons completely devoid of myelin). Based on the g-ratio (see "Materials and methods" section, we also examined the frequency of **c** optimally myelinated and **d** demyelinating axons. Data are presented as the mean ± SEM. *P ≤ 0.05; paired *t* test

A quantification of the electron micrographs revealed that axolytic fibers, visually identified based on the absence of discernable neurofilaments, presence of swollen mitochondria, and unraveling myelin (Fig. 5b, c), were significantly (*p* = 0.042) less common in +EAE/+AAVCre optic nerves (2.573 ± 0.4507; *n* = 11) than in their −AAVCre contralateral counterparts (4.136 ± 0.8918; *n* = 11; Fig. 6a). Demyelinated fibers, visually identified based on the presence of an intact axon but devoid of myelin, were also less frequent (*p* = 0.0470) in +EAE/+AAVCre (12.28 ± 2.716; *n* = 11) than in their −AAVCre contralateral counterparts (19.06 ± 2.813; *n* = 11; Fig. 6b).

In the remaining fibers that were not visually identified as either axolytic or demyelinated, myelin pathology was quantified by using the g-ratio [21], dividing the axonal diameter by the diameter of the axon plus myelin sheath. The optimal g-ratio in the optic nerve in naïve −EAE/−AAVCre flox mice was established at 0.77 ± 0.060 S.D. (*n* = 3) which was similar to wild-type C57BL/6 at 0.76 ± 0.070 S.D. (*n* = 6). A conservative margin of ±3 standard deviations from the mean of normal −EAE/−AAVCre flox mice was used as the cutoff to assign a diagnosis of demyelinating (< 0.59) or remyelinating (> 0.95), with intermediate g-ratios being considered as optimally myelinated. Using these parameters, we found no remyelinating fibers in any group (not shown), while all (100%) of the quantified axonal fibers in non-EAE animals were optimally myelinated. In the EAE-treated groups −AAVCre mice had significantly fewer (*p* = 0.0427) optimally myelinated fibers (87.08 ± 3.669; *n* = 11) than +AAVCre mice (92.72 ± 2.283; *n* = 11; Fig. 6c). None (0%) of the non-EAE had demyelinating fibers. In the +EAE/+AAVCre group, the proportion of demyelinating axons was found significantly (*p* = 0.0311) reduced (7.308 ± 2.276; *n* = 11) relative to their −AAVCre contralateral counterparts (13.17 ± 3.632; *n* = 11; Fig. 6d).

## Discussion

Myelin, in addition to its electrical insulating properties, is essential to the organization of the nodes of Ranvier which ensure the efficient propagation of the action potential by saltatory conduction [24, 25]. In demyelinating diseases, including MS, myelin loss leads to a disruption of the molecular cues and anchors that maintain the integrity of the nodes and, in turn, the membrane proteins of the axons become displaced and their expression dysregulated. Among these proteins, the voltage-gated sodium channel Nav1.6 is believed to play an important role in the axonal degradation that eventually follows demyelination or cycles of demyelination. Interestingly, demyelination does not only cause the dispersal of the pre-existing channels that were present at the nodes of Ranvier but in fact increases the density of the Nav channels in animal models [26–28] and in MS lesions [29]. The co-localization of Nav1.6, the Na^+^/Ca^2+^ exchanger (NCX), and markers of axonal injury has led to the hypothesis that the persistent influx of Na^+^ through Nav1.6 channel in MS, and in the EAE animal model of MS, causes the NCX to operate in reverse, leading to the toxic accumulation of intracellular Ca^2+^ ions that results in cell death and axonal degradation [10, 22, 30, 31]. Alternatively, Nav1.6 has been implicated in the release of Ca^2+^ from intra-axonal stores [32]. The role of Nav1.6 in degeneration has been difficult to verify directly since *Scn8a*/Nav1.6-null mice (i.e., whole-body mutants) die around 21 days post-partum, which make them unsuitable for EAE induction [33, 34]. We chose to target *Scn8a* specifically in the retina and optic nerve for studying demyelination and axonal loss since optic neuritis is prominent and well-characterized in EAE mice [35, 36]. We targeted *Scn8a* in a single optic nerve by intravitreal injection of an adeno-associated virus harboring the Cre recombinase and enhanced GFP (eGFP) genes under the control of the CMV promoter (AAV2-Cre-GFP) in mice homozygous for the floxed *Scn8a* allele [15]. *Scn8a* was targeted in retinal ganglion cells by using the serotype 2 variant of the adeno-associated virus (AAV2), which has been shown to transduce approximately 34% of the RGC population when administered by intravitreal injection, although it should be noted that in this study by Smith and Chauhan [37], the DCX promoter was used while we have used the CMV promoter. Subsequently, to induce EAE, we used MOG_35–55_ as the antigen since it induces chronic monophasic EAE in C57BL/6 mice [16, 38]. The contralateral eye was used as an internal control, which was either injected with an AAV2 vector expressing GFP alone (AAVGFP) or left non-injected. This approach allows us to compare Cre-targeted and control samples that are exposed to the same disease micro-environment; a significant advantage since the EAE disease severity can vary considerably between animals [3]. Furthermore, the absence (*Il6* and *Ifng*) or near absence (*Gfap*) of expression in the non-EAE + AAVCre control strongly suggests that the effects observed in the +EAE/+AAVCre mice are indeed due to the inactivation of *Scn8a*/Nav1.6 and not to a non-specific effect of the AAV2 virus.

Inner-retinal cell targeting was confirmed by eGFP expression, as detected by CSLO in vivo imaging [37] which allowed us to longitudinally track the number of Nav1.6 knockout cells. Following injection, the number of RGCs targeted increased in a linear fashion until day 78 consistently with the observations of [37]. Immunostaining against RBPMS of flat-mounted retinas from 41 days post-EAE induction revealed a massive loss of RGCs, in accordance with previously reported RGC loss in EAE-associated optic neuritis [35, 39]. The co-localization of the remaining morphologically normal-appearing RBPMS-positive RGC cell bodies with GFP strongly suggests that the elimination of Nav1.6 within neurons promotes cell survival. Real-time quantitative assessment of *Rbpms* and *Scn8a* retinal expression revealed that within each animal, the +AAV2Cre retina expressed less *Scn8a* and more *Rbpms* than the retina from the non-injected or +AAVGFP contralateral eyes. Furthermore, when compared as groups, the +AAV2Cre eyes differed significantly from the non-injected and +AAVGFP contralateral eyes. Taken together, these observations corroborate the hypothesis that Nav1.6 exacerbates RGC death in EAE.

The main trigger of MS is believed to be an inflammatory autoimmune response within the CNS that causes tissue destruction including demyelination and axonal damage [3]. Inflammation in the CNS is generally initiated by microglia, the resident macrophages, and other immune cells that can cross the blood-brain barrier (BBB), such as macrophages, T cells, and B cells that exacerbate the inflammatory response [40]. Furthermore, this inflammation can lead to the disruption of BBB and increase the infiltration of immune cells into the CNS [41–43]. Quantitative RT-PCR expression analyses revealed that the reduction of *Scn8a* expression in the +AAVCre eye versus the control contralateral eye was associated with decreased retinal expression of pro-inflammatory cytokines *Il6* (IL-6) and (*Ifng)*, robust indicators of inflammation. Antibody blockade studies of IL-6 and knockout studies of IFN-γ have revealed that these cytokines are implicated in the induction of EAE [44–46] Furthermore, a notable reduction within individual mice of reactive gliosis as indicated by a marker of fibrillary acidic protein (*Gfap*) [47], was also observed in +AAVCre eye compared to the fellow eye. A recent study by Wilmes et al. [48] showed that in acute and chronic phases of EAE, a glia scar is formed by reactive astrocytes. It has been shown that increased expression of GFAP in Müller cells is an indicator of the activation of astrocytes and the loss of RGC, which may be triggered by inflammation and apoptosis [47]. Several underlying mechanisms that involve the interaction between the immune cells and the neurons are believed to impact the development of the disease and Nav channels may play a central role in this interaction due to their expression in both types of cells [22, 49]. Nav1.6, in particular, is expressed in non-neuronal cells, such as astrocytes, microglia, and macrophages as well as in invasive cancer cell lines, where they are believed to contribute in the ability of these cells to mobilize by activating the actin cytoskeleton leading to the formation of podosomes and invadopodia [50–53]. In the context of this study, the presence of Nav1.6 in non-neuronal cells raises the question as to whether our observations result from deleting Nav1.6 in RGCs or if the presence of +AAVCre/Nav1.6-null non-neuronal cells might be impacting the results. Of the total number of retinal cells transduced by AAV2 approximately 65% are ganglion cells, while approximately 9% are Müller cells [54] and in the EAE chronic phase (41 days following induction) all the observed +AAVCre cells also stained positively for RBPMS (Fig. 2d). Therefore, while we cannot completely eliminate the possibility that cells other than RGCs, such as Müller cells, might contribute to the reduction in retinal inflammation, we believe this contribution to be minimal. Nav1.6 expressed in neurons, therefore, appears to promote inflammation in EAE, although it is unclear if this is due to the increased axonal degeneration or to a more direct influence of Nav1.6 on immune cells.

A prominent feature of the optic neuritis associated with EAE and MS is the thinning of the retinal nerve fiber layer and loss of axons which can result in permanent vision disruptions [39]. Even in axons that survive demyelination after the inflammation resolves only limited remyelination usually occurs causing a decrease in the action potential conduction and nerve atrophy [55]. We observed by histological staining that +AAVCre optic nerves had fewer infiltrating immune cells than EAE+ optic nerves from −AAVCre eyes and that the amount of infiltrating macrophages, as estimated by flow cytometry (F4–80^+^ CD11b^+^), was reduced in the optic nerve from +AAV2Cre optic nerves. Horstmann et al. [56] showed that at day 60, during the late stage of EAE, an increased microglial cell response was associated with increased RGCs loss and increased cell infiltration in the optic nerve, which was consistent with our findings.

Ultrastructural analysis of axonal damage in optic nerve revealed axonal degeneration, accompanied by degeneration of the myelin sheath, which is the main feature of the disease. Our observation showed that the optic nerves from +AAVCre eyes have decreased demyelination and fewer axolytic fibers compared to the control fellow eye. This is consistent with O’Malley et al. [57] who found that sodium channel β subunits knockout mice show reduced axonopathy following induction of EAE. Moreover, O’Malley et al. [57] showed that the lack of *Scn2B* (β2) subunit in mice reduces the severe clinical symptoms and axonal degeneration in EAE. This protective effect is independent of the immune response and it was attributed to the downregulation of Nav1.6, thus reducing the harmful effect of Ca2^+^ accumulation in axons.

Nav channel involvement in the etiology of MS has long been recognized. For example, pharmacological treatment using broad-spectrum blockers including phenytoin, lidocaine, carbamazepine, flecainide, safinamide, and TTX have shown efficacy in animal models of anoxia and NO-mediated damage anoxia and in EAE mice [30, 58–62]. However, the efforts to target Nav channels for the treatment of degenerative diseases in humans have faced challenges due to the complex structure of these channels, the lack of selective pharmaceutical inhibitors, and their broad expression on neuronal and non-neuronal cells. Clinical trials conducted with lamotrigine [63] and phenytoin [64, 65] have yielded equivocal results indicating that more research is required to clarify how blocking Nav channel isoforms expressed in excitable and non-excitable cells impacts disease progression [66]. 4,9-Anhydrotetrodotoxin (4,9-ah-TTX), a metabolite of TTX, blocks Nav1.6 in the nanomolar range with minimal effect on other TTX-sensitive channels [67, 68]. Hargus et al. [69] have shown that 4,9-ahTTX selectively blocks Nav1.6 but not Nav1.2 currents and was able to suppress neuronal hyperexcitability in a mouse model of epilepsy. Recently, the microRNA miR-30b-5p was shown to downregulate Nav1.6 in a rat model of neuropathic pain and was used to attenuate neuropathic pain induced by oxaliplatin [70]. As such, new blocking or downregulation strategies for Nav1.6 may soon become available and could offer interesting therapeutic options for MS.

## Conclusion

The molecular mechanism of axonal degeneration in MS is highly complex and involves several neurological and immunological elements. Here, we demonstrate for the first time that a “null” genetic lesion in neuronal Nav1.6 has a neuroprotective effect in vivo. Our results corroborate and extend previous findings that Nav1.6 is a promoter of neuronal degeneration and inflammation in EAE [51], suggesting that it plays a corresponding role in MS and possibly in other degenerative neurological diseases. Our results suggest that downregulating or blocking Nav1.6, specifically on neuronal cells, would be neuroprotective and could widen the window for other therapies. However, based on its ubiquitous localization at axon initial segments and nodes of Ranvier and on the phenotype displayed in *Scn8a* mouse mutants such as juvenile lethality in null mice and severe ataxia in channel gating mutants [71], Nav1.6 plays an essential physiological role and targeting this isoform involves inherent risks. Nevertheless, there is evidence that other isoforms, such as Nav1.2, can effectively compensate for the loss of Nav1.6. During postnatal development, Nav1.2 is normally expressed along the optic nerve to be replaced later, at advanced stages of development, with Nav1.6 [72, 73]. Studies have shown that Nav1.2 plays a compensatory role for partial loss of Nav1.6 and may be able to conduct signals in demyelinated axon [11, 74]. As such, a mechanistic basis upon which the targeting of Nav1.6 may provide an effective treatment exists and it will be of primary interest to study the compensatory role of this channel in the context of EAE and MS.

## Supplementary information

**Additional file 1: Figure S1.** Clinical score and weight progression of Scn8a ‘floxed’ and wild-type C57BL/6 mice. Eight to ten-week-old female mice were immunized with myelin oligodendrocyte glycoprotein peptide (MOG_35–55_) with complete Freund’s adjuvant and pertussis toxin. The progression of the clinical score (A) and weight profile (B) are similar for *Scn8a* homozygous ‘floxed’ (Scn8aflox/flox on C57BL/6 genetic background) mice that were used in this stud and for control wild type C57BL/6 mice (*n* = 10 for each group).

## Data Availability

The datasets used and analyzed during the current study are available from the corresponding author on reasonable request.

## Abbreviations

AAV2: Adeno-associated virus, serotype 2
BBB: Blood-brain barrier
CFA: Complete Freund’s adjuvant
CMV: Cytomegalovirus
CNS: Central nervous system
CSLO: Confocal scanning laser ophthalmoscopy
EAE: Experimental autoimmune encephalomyelitis
MOG: Myelin oligodendrocyte glycoprotein
MS: Multiple sclerosis
PFA: Paraformaldehyde
PTX: Pertussis toxin
qRT-PCR: Quantitative real-time polymerase chain reaction
*Scn8a*: Gene that encodes the Nav1.6 α subunit
