# Supplementary figures and images for: Nav1.6 promotes inflammation and neuronal degeneration in a mouse model of multiple sclerosis
Source: J Neuroinflammation. 2019 Nov 13;16:215. doi: 10.1186/s12974-019-1622-1 (PMC6852902; doi:10.1186/s12974-019-1622-1)

ALRASHDI ET AL. - SUPPLEMENTARY FIGURE 1

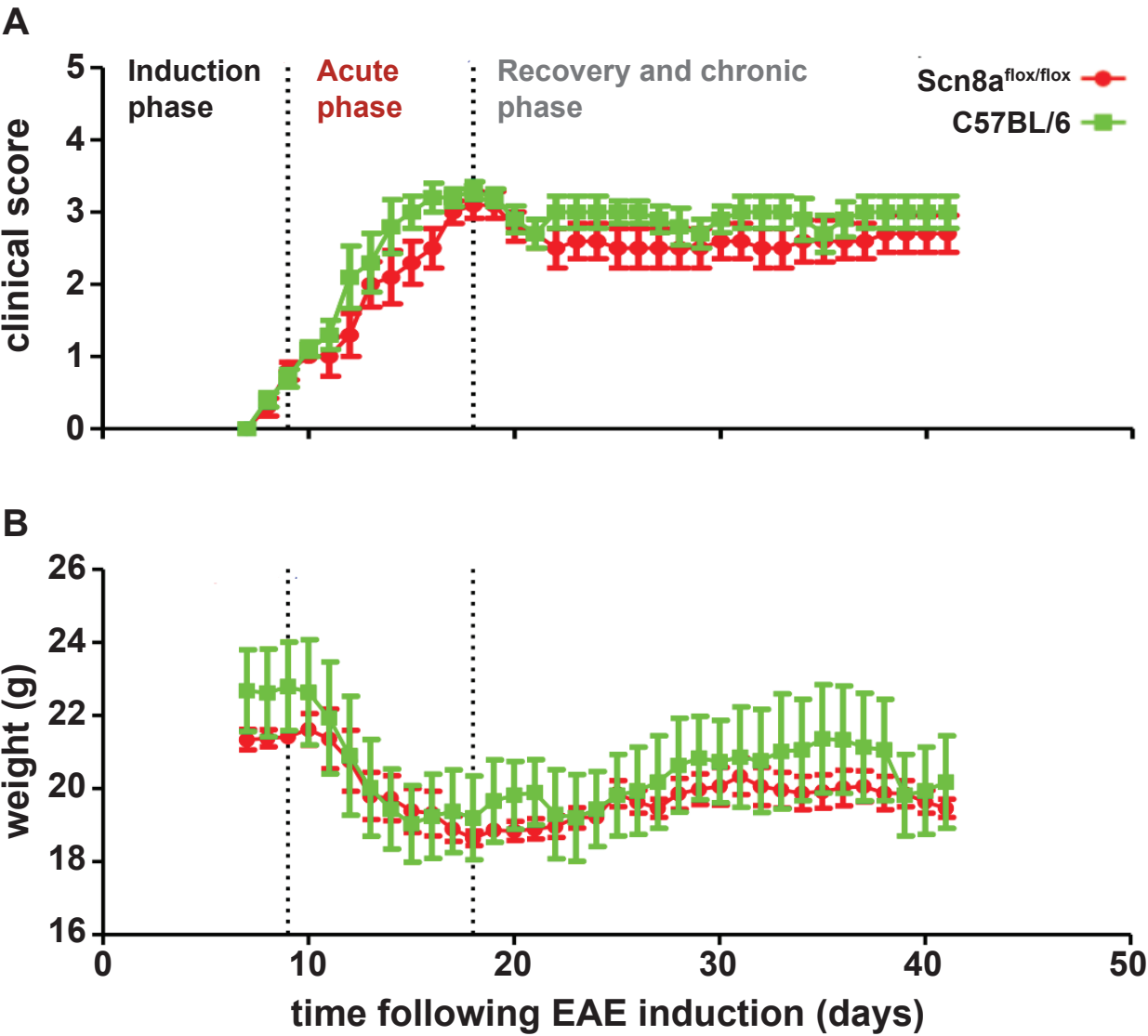

Supplement: Supplementary file 1 — Additional file 1: Figure S1. Clinical score and weight progression of Scn8a ‘floxed’ and wild-type C57BL/6 mice. Eight to ten-week-old female mice were immunized with myelin oligodendrocyte glycoprotein peptide (MOG35–55) with complete Freund’s adjuvant and pertussis toxin. The progression of the clinical score (A) and weight profile (B) are similar for Scn8a homozygous ‘floxed’ (Scn8aflox/flox on C57BL/6 genetic background) mice that were used in this stud and for control wild type C57BL/6 mice (n = 10 for each group). [file 12974_2019_1622_MOESM1_ESM.pdf]
